# An examination of the predictors of change in BMI among 38 026 school students in Makkah, Saudi Arabia

**DOI:** 10.1093/inthealth/ihae029

**Published:** 2024-04-05

**Authors:** Mohammed Banany, Klaus Gebel, David Sibbritt

**Affiliations:** School of Public Health, Faculty of Health, University of Technology Sydney, 235 Jones St, Ultimo, NSW 2007, Australia; School of Public Health, Faculty of Health, University of Technology Sydney, 235 Jones St, Ultimo, NSW 2007, Australia; Prevention Research Collaboration, Charles Perkins Centre, School of Public Health, the University of Sydney, Camperdown, NSW 2006, Australia; School of Public Health, Faculty of Health, University of Technology Sydney, 235 Jones St, Ultimo, NSW 2007, Australia

**Keywords:** BMI, children, obesity, overweight, school-based intervention

## Abstract

**Background:**

The prevalence of childhood obesity has substantially increased in the Gulf Cooperation Council countries, including Saudi Arabia. The Rashaka initiative is a Saudi national school-based multicomponent intervention that was introduced in the school year 2016–2017 to address childhood overweight and obesity. This study aims to examine the effect of the Rashaka initiative on students’ body mass index (BMI) for two academic years (2016–2017 and 2018–2019) and to analyse predictors of BMI change.

**Methods:**

Secondary data for this pre–post study was provided by the Ministry of Health for 38 026 students from 89 intermediate and secondary schools that implemented the initiative in Makkah City, Saudi Arabia. It was analysed using non-parametric tests and multiple regressions at a 5% level of significance.

**Results:**

Over 2 y of implementation, BMI was reduced significantly across the schools (p < 0.001). Based on the regression modelling, school gender and education stage were found to be the only significant predictors of BMI change. Girls and intermediate schools had greater BMI reductions than boys and secondary schools (p < 0.001 and p = 0.031).

**Conclusions:**

This study provides tentative evidence for the effectiveness of the Rashaka intervention in Makkah City. In addition, our study has identified that the Rashaka initiative may require modification to improve its effect on boys and students in secondary schools.

## Introduction

Obesity among children and adolescents is a public health priority in developed and developing nations.^1^ In the past few decades, the Gulf Cooperation Council (GCC) countries (Saudi Arabia, United Arab Emirates, Kuwait, Bahrain, Oman and Qatar) have witnessed substantial sociocultural and lifestyle transitions, contributing to the adoption of high-calorie diets, physical inactivity and increased sedentary behaviours, leading to a drastic increase in childhood obesity.^2,3^ In Saudi Arabia, a study conducted in 2022 found that 25.7% of school-aged children were considered overweight or obese.^4^

Childhood obesity is a preventable disease requiring effective strategies that target environmental and sociocultural factors associated with dietary behaviours and physical activity.^5–7^ Schools are considered to be ideal venues for implementing weight-related interventions, as students spend a significant amount of their time there and because of school meal programs, physical education classes, facilities and other school environmental factors.^8–13^ School food services that provide healthy and nutritious alternatives to energy-dense foods that are low in nutrients have been associated with lower obesity rates.^9,14,15^ A systematic review found that in mainland China, school physical education programs combined with physical activity facilities were effective in preventing and reducing overweight and obesity.^9^ However, a study conducted in Australia by Barnes et al.^16^ concluded that interventions for physical activity, nutrition or both were ineffective on the children's weight or their quality of life and another study in China had a similar conclusion, where school-based interventions showed a limited efficacy on childhood obesity.^17^

In Saudi Arabia, the Rashaka initiative is a national, school-based program that was launched in 2017 to address the rising rates of obesity among schoolchildren and adolescents.^18^ The Rashaka initiative is comprised of multicomponent interventions focusing on the school environment, which refers to the facilities and equipment within the school's borders and fences, including buildings, playgrounds, canteen and eating spaces during recess. In addition, the initiative interventions also focus on students’ social and lifestyle factors in reducing their body weight. This study aims to evaluate the effectiveness of the Rashaka initiative in reducing overweight and obesity among school students. It also aims to examine predictors of change in body mass index (BMI) to inform policymakers and other stakeholders for better evidence-based obesity interventions in schools.

## Methods

### Study design and population

This pre–post study analysed data collected from 38 026 male and female students (age range 13–18 y) from 89 intermediate and secondary schools in Makkah City, Saudi Arabia.

### Sample selection and source of data

All intermediate and secondary schools in Makkah City (n = 509) were invited to participate in the Rashaka initiative (cluster sampling) and 231 schools (about 45%) took part in the intervention. We received secondary data from the Saudi Ministry of Health for 89 of these schools with a total sample size of 38 026 students.

### Outcome variables

The dataset included the height and weight of students collected in the school years 2016–2017 and 2018–2019. Height was measured to the nearest 0.1 cm and weight to the nearest 100 g by trained school health counsellors. BMI was then calculated by dividing weight in kilograms by height squared in meters. The impact of the initiative was examined at the school level using the change in BMI. For each school, the change in BMI was calculated as the difference between the mean BMIs of 2016–2017 and of 2018–2019. Other school-level data included school educational stage (intermediate [13–15 y of age], secondary [16–18 y of age]), gender (girls, boys), school type (public, private) and geographical region (East, West, Centre, North, South).

Twelve school environment–related actions and activities in the Rashaka initiative were categorized into four main factors. The first factor (F1) is school food services, such as serving a variety of healthy choices of foods and drinks, prohibiting the provision of high-calorie drinks and foods or unhealthy snacks. The second factor (F2) is the school facilities, such as creating additional safe play areas for physical activity and the establishment of a school health clinic. The third factor (F3) is about school policies, which include regular assessment of students’ sedentary behaviour and the creation of the Rashaka team, which comprises the school principal, school health counsellor, some students and some parents who volunteer and are responsible for implementing the initiative's interventions and monitoring its progress. In addition, F3 consists of a daily assessment of the school canteen using a checklist, applying the health requirements for school canteens and periodic scheduled school visits from health professionals. The last factor (F4) is about school health-promoting activities such as a monthly healthy breakfast and the Rasheeq student competition, which involves rewards such as certificates or trophies for students who lose weight, engage more in physical activity, eat a healthy diet and decrease their sedentary behaviours. Using a relevant form/checklist, each of the 12 factors were assessed and implementation reported (coded as a 1 if implemented and a 0 if not). F1 and F2 included three subfactors, F3 included four subfactors and F4 included two subfactors. The total score was calculated by adding F1–F4 for both 2016–2017 and 2018–2019. The change in the total score was derived by calculating the difference in the total scores between 2016–2017 and 2018–2019. For each of the four main factors, the change (F1c, F2c, F3c, F4c) was calculated between 2016–2017 and 2018–2019.

### Statistical analysis

Statistical analysis was conducted using the Statistical Package for the Social Sciences version 28.0 (IBM, Armonk, NY, USA). Different school characteristics were used to present the frequencies and the percentages of the study participants. The normality distribution of BMI was examined using the Kolmogorov–Smirnov test. Accordingly, bivariate analysis was undertaken, which included the Wilcoxon signed-rank test to assess the impact of the intervention across the 2 y of follow-up and the Mann–Whitney U test and Kruskal–Wallis test, where appropriate. Backward stepwise linear regression modelling was carried out to determine the most important significant predictors of change in BMI. The adjusted R^2^ was used to ascertain the model fit. Significance was defined as a p*-*value <0.05.

## Results

### Sample description

Data were collected from 38 026 students from 89 schools, where more than three-quarters of them were girls’ schools (68 schools). The sample consisted of primarily public schools (81 schools [91%] and the participants were almost equally distributed between secondary (46 schools) and intermediate (43 schools). Twenty-one schools each were from the North and West regions, 14 each from the East and Central regions and 19 from the South region (Table 1).

**Table 1.** Comparison of participants’ median change in BMI.

		Change in BMI	
Variables	Schools, n (%)	Median (IQR)	p-Value
School gender			<0.001*,^a^
Girls	68 (76.4)	2.52 (1.11)	
Boys	21 (23.6)	0.44 (4.39)	
School education stage			0.004*,^a^
Intermediate	43 (48.3)	2.70 (0.93)	
Secondary	46 (51.7)	1.96 (1.49)	
School type			0.966^a^
Public	81 (91)	2.35 (1.55)	
Private	8 (09)	2.02 (3.54)	
Geographical region			0.266^b^
North	21(23.6)	2.11 (2.80)	
East	14 (15.7)	2.34 (1.10)	
South	19 (21.3)	2.50 (1.09)	
West	21 (23.6)	2.50 (2.33)	
Centre	14 (15.7)	2.66 (1,67)	

* *P*-value is significant at <0.05.

^a^Mann–Whitney U test.

^b^Kruskal–Wallis test.

### Changes in BMI

Overall, there was a significant reduction in BMI between 2016–2017 (median 22.54) and 2018–2019 (median 19.84) (p < 0.001). The BMI change was calculated for each school and the median comparisons are provided in Table 1. As presented in Table 1, girls’ schools (median 2.52 [interquartile range {IQR} 1.11]) had more significant reductions in BMI compared with boys’ schools (median 0.44 [IQR 4.39]) (p<0.001). Intermediate schools (median 2.70 [IQR 0.93]) also had more significant reductions in BMI compared with secondary schools (median 1.96 [IQR 1.49]) (p = 0.004). However, no significant differences were shown in the median change in BMI across all the other school characteristics (school type and geographical region).

### Predictors of change in BMI

Further analysis was undertaken using multiple linear regression modelling to examine environmental aspects of the schools predicted BMI change. The initial model included the total score change as well as school-level characteristics, including education stage, school type and gender. A backward stepwise approach was then used to determine the most parsimonious model for predicting change in BMI, with the final model presented in Table 2.

**Table 2.** Linear regression model to predict change in BMI.

			95% CI			
Predictors	Categories	Coefficient (β)	Lower	Upper	p-Value*	Adjusted R^2^^a^
Constant		5.051			<0.001*	0.34
School type	Public (ref)					
	Private	1.106	−0.040	2.252	0.058	
School gender	Girls (ref)					
	Boys	−2.675	−3.454	−1.896	<0.001*	
Education stage	Intermediate (ref)					
	Secondary	−0.719	−1.369	−0.068	0.031*	

Ref: reference category.

* P-value is significant at <0.05.

a Adjusted for school type, school gender and education stage.

As shown in Table 2, the only statistically significant predictors of change in BMI were school gender and education stage. That is, on average, the decrease in BMI was significantly lower for students attending secondary schools compared with those attending intermediate schools (β = −0.719, p = 0.031) and in boys’ schools compared with girls’ schools (β = −2.675, p < 0.001).

## Discussion

This large longitudinal study found that the students’ mean BMI decreased significantly between two academic years (2016–2017 and 2018–2019) across all the schools. One possible reason for this decrease in mean BMI was the implementation of the Rashaka initiative over this period. In a situation where multiple components were included in the initiative and weight was identified as a performance indicator,^18^ a change in BMI may be attributed to the impact of the intervention program. However, due to the lack of control groups, we cannot exclude the possibility that the observed change in BMI may also be attributed to natural body changes that occur during puberty.^19,20^

Interestingly, there was a significant difference in the change in BMI based on gender and education stage. In terms of gender, the decrease in girls’ mean BMI was greater than that of the boys. Gender differences in students’ weight, BMI or weight-related dietary behaviours and physical activity have been examined in several studies globally. For instance, while in Spain, a school-based physical activity intervention did not show any differences in outcomes between boys and girls,^21^ while in Argentina, girls were reported to have more improvement in their dietary intake compared with boys.^22^ However, a recent systematic review found no significant differences between boys and girls in any of the body composition measures, including weight, BMI, BMI z-score, percentage body fat and waist circumference.^23^ In the Gulf region specifically, Choudhury et al.^24^ reported no gender differences in BMI or other body composition measures. Similarly, in a school-based intervention conducted in Kuwait, there was no significant gender difference in participants’ BMI change.^25^ Given that our findings conflict with most others on the topic, it would appear that further investigation on gender differences is warranted. Interestingly, in 2017, Al Eid et al.^18^ stated that one of the 10 challenges in the Rashaka initiative was to provide female-specific sports classes.

A further interesting finding is the change in BMI across education stage. Our study found that reductions in students’ BMI were significantly lower for secondary school students than those attending intermediate schools (β = −0.719, p = 0.031). This difference might be due to high or secondary schools potentially having fewer restrictions regarding the availability of high-energy foods and sugar-sweetened beverages than intermediate schools. However, no studies were found that compared BMI change between intermediate or middle schools and secondary or high schools. Therefore, governance for obesity-related policies promoting a healthy dietary environment should be strengthened in secondary schools to enhance the intervention effectiveness in promoting changes in students’ BMI.

Another interesting finding from the regression modelling was that the school environment did not predict BMI change. This may be due to the type of data collected from the schools, with binary answers for whether an aspect of the environmental intervention was implemented or not. Evaluations of school interventions have shown that school environmental factors can have an impact on student weight management and the promotion of a healthy lifestyle.^26–28^ In contrast, Habib-Mourad et al.^12^ evaluated a nutritional intervention in Lebanon and emphasized that they were unable to change the school environment, such as physical conditions, or the availability of healthy food choices, otherwise they could have achieved more meaningful improvements in students’ diets.

### Strengths and limitations

This study had some limitations to consider when interpreting our findings. The 12 factors of the Rashaka initiative were evaluated by checking whether they were implemented or not, limiting the understanding of the quantity and quality of the relevant interventions carried out within the program. While 231 schools from Makkah City took part in the intervention, we only received data from 89 schools from the Saudi Ministry of Health. Therefore, we cannot exclude the possibility that this might have introduced selection bias. Furthermore, we could not follow students who relocated to another school/city. One of the strengths of this study is its large sample size. A further strength is the analysis of multiple school environmental factors applied in the Rashaka initiative. As well, the follow-up time of 2 y had the potential to capture meaningful changes in BMI^29^ compared with other studies in the Gulf region, which only had follow-up times of 1–6 months.^30–31^

## Conclusions

This study provides some evidence that the implementation of the Rashaka initiative may have resulted in a reduction of the students’ BMI. The differences observed across gender and education stage warrant further investigation of the Rashaka initiative's implementation to determine how it can be improved to better assist boys and students attending secondary schools in reducing their BMI. Therefore, to reduce the change in the BMI gap across gender and education stage, students at boys’ and secondary schools should be educated more about healthy lifestyles and obesity. Additionally, obesity-related policies and interventions should be promoted and supervised in such schools in the future to overcome obesity consequences. Furthermore, longer-term monitoring of the schools that implemented the intervention would be desirable to better understand the impact and outcome of the intervention and to identify aspects that need to be improved.

## Data Availability

The data underlying this article were provided by the Saudi Ministry of Health by permission. Data will be shared upon request to the corresponding author with permission from the Ministry of Health, Saudi Arabia.
